# Design of a miniaturized ultrawideband and low scattering antipodal vivaldi antenna array

**DOI:** 10.1038/s41598-021-92051-z

**Published:** 2021-06-14

**Authors:** Jia Liu, Chengxiang Xu, Hang Yu, Jianxun Su

**Affiliations:** State Key Laboratory of Media Convergence and Communication, Communication University of China, Beijing, 100024 China

**Keywords:** Electrical and electronic engineering, Electronic devices

## Abstract

This paper presents a miniaturized ultra-wideband (UWB) antipodal Vivaldi antenna (AVA) array with low-scattering characteristics integrated a hybrid diffusive-absorptive metasurface. Periodic elliptical slots at the outer edges and a dielectric lens are utilized for antenna element to improve performances including miniaturized size, wide bandwidth, and high gain. The optimized element is fabricated and measured, the results demonstrate that the − 10 dB impedance bandwidth is 4.5–50 GHz with a ratio bandwidth (*f*_H_/*f*_L_) of 11.1:1, and the maximum gain at 35 GHz is 12.7 dBi, which are in good agreement with simulation. By loading an optimized Minkowski-shaped metasurface as the ground reflector, which combines the multielement phase cancellation (MEPC) and EM absorption technology, the 4 × 4 array realizes a low radar cross section (RCS) without the radiation performance degradation. Simulated and measured results show that the proposed low-scattering array has a 10-dB RCS reduction band ranging from 5 to 50 GHz at normal incidence for both polarizations. Furthermore, the array structure shows extremely low-observable capability, which is larger than 15 dB of the RCS reduction from 7.1 to 50 GHz with a ratio bandwidth of 7.0:1. The results verify the feasibility of improving the performance of antenna and the UWB low-scattering functionality.

## Introduction

With the continuous escalation of information warfare, in the limited space of the combat platform, electronic systems with multiple functions need to be integrated to complete different tasks. The development of antenna arrays with miniaturization, ultra-wideband (UWB), high-gain, and low-scattering characteristics have attracted increasing attention. Antenna is a main contributing source to the overall radar cross section (RCS) of the military platform. Thus, reducing its RCS while maintaining the radiation properties is crucial to military platform.

Antipodal Vivaldi antenna (AVA), which is a kind of end-fire traveling-wave antenna with UWB, high efficiency, small lateral dimension, easy manufacturing process, and so on, has been widely used in wireless and radar military applications^1–3^. However, there are some drawbacks^4^ of the conventional Vivaldi antennas including the low-end bandwidth limitation, large size, and low gain. For improving the radiation performance of the AVA, such as gain, front and back ratio, bandwidth, radiation pattern, polarization and operating frequency range, the general method is to modify the physical geometry of the antenna, or add such as slot, corrugation, fractal shapes, dielectric lens to eliminate the undesirable effects of beam squint and cross polarization^5^. Different kinds of slots, such as the elliptically shaped strip conductors^6^ the exponential slot edge^7^, multiple slots with varied lengths^8^, etc., have been reported to realize impedance matching and high directivity. In addition, dielectric lens^9–12^, which is also called as director, is also utilized to enhance end-fire radiation performances at higher frequency of the AVA.

With the development of stealth and detection technologies, since the antenna array generally contribute significantly to the RCS of a military target, a lot of methods^13–17^ have been proposed to obtain low-scattering feature. A low scattering microstrip antenna array has been designed by replacing the conventional metallic ground to a coding artificial magnetic conductor (AMC) ground^14^. In order to avoid the influence of the AMC ground on the radiation performance, the operating frequencies of scattering and radiation are different, which will limit its application. Characteristic mode cancellation (CMC) method has been developed to design a low-RCS characteristic antenna both in band and out of band^15^. To broaden the bandwidth, polarization conversion metasurface^16^, phase gradient metasurface^17^, and frequency selective surface^18^ were placed over the array antenna to obtain diffuse scattering property. However, the RCS reduction operating band of most work^13–18^ are limited, meanwhile, few literatures for UWB antenna array with low-scattering characteristics design has been published.

This paper presents a low-scattering AVA array with a 10 dB impedance bandwidth from 4.5 to 50 GHz and the 15 dB RCS reduction nearly covering the whole operating band. First, a miniaturized UWB AVA antenna element is designed using a serious of elliptical slots at the outer edges and a dielectric lens loading to miniaturize size, widen bandwidth, and enhance gain. Then, a novel hybrid diffusive-absorptive method, based on multielement phase cancellation (MEPC)^19^ and EM absorption technology, is proposed to realize a low-observable metasurface with more than 15 dB RCS reduction over UWB. By replacing the optimizing metasurface to the array ground reflector, a miniaturized UWB AVA array with the UWB low-scattering property is presented without the radiation performance degradation. Simulation and measurement results demonstrate that our proposed antenna array can operate over UWB with low observability.

## Design and theory of AVA array

**Figure 1:**
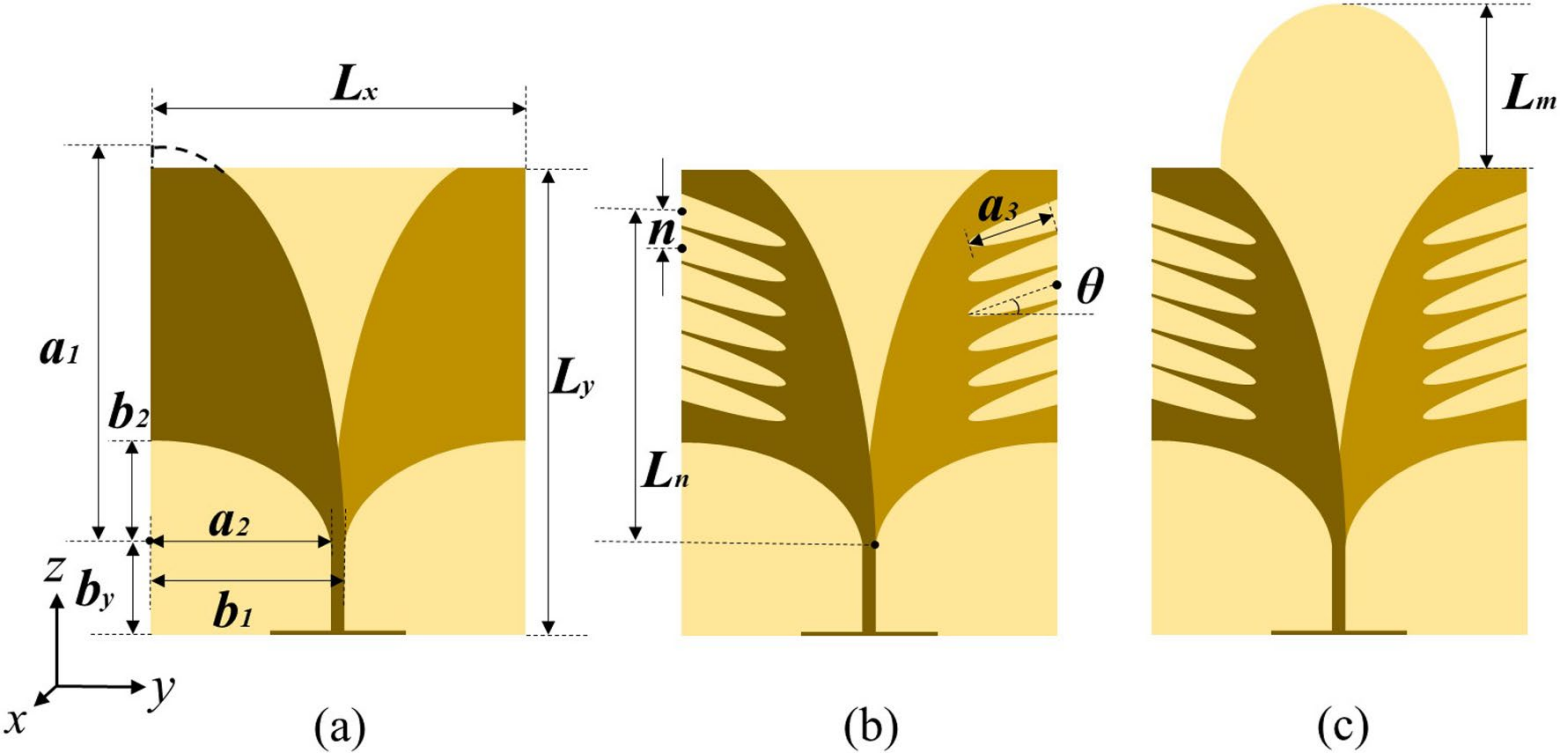
Structure of **(a**) reference AVA, (**b**) slotted AVA and (**c**) lens-loaded AVA (*L*_*x*_ = 30 mm, *L*_*y*_ = 40 mm, *a*_*1*_ = 33.56 mm, *b*_*1*_ = 15.5 mm, *a*_*2*_ = 14.5 mm, *b*_*2*_ = 8.8 mm, *b*_*y*_ = 8.5 mm, *n* = 3 mm, *a*_*3*_ = 8.5 mm, *θ* = 20°, *L*_*n*_ = 19 mm, *L*_*m*_ = 15 mm).

As illustrated in Fig. 1a, the reference AVA, we used as the prototype antenna, is composed of two upper and lower designed elliptical metal radiating patches loaded on both sides of the dielectric substrate of Rogers RO4003C (ε_r_ = 3.38) with a thickness of 0.508 mm. The dimension of the entire antenna is 30 mm × 40 mm.

**Figure 2:**
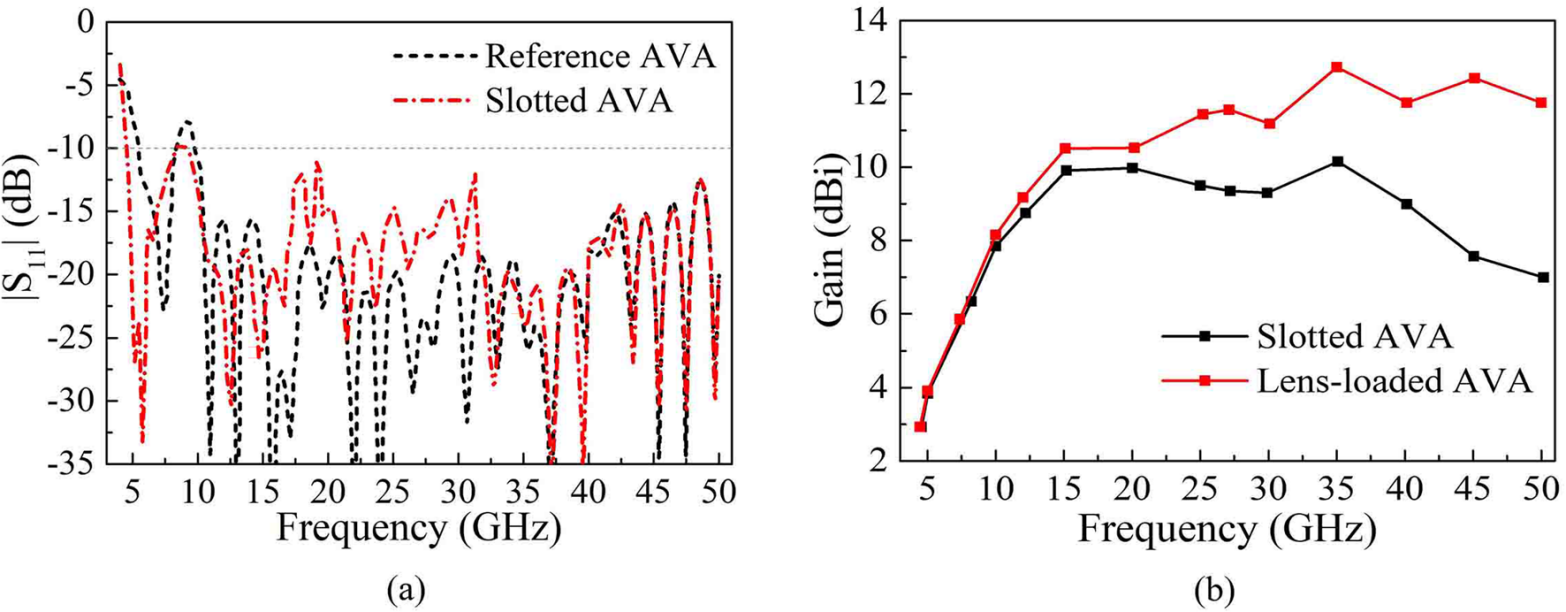
Comparison of (**a**) |S_11_| of reference AVA and slotted AVA and (**b**) gain of slotted AVA and lens-loaded AVA.

However, it is obviously displayed in Fig. 2 that the reference AVA has a poor impedance matching in low frequency range of 8–10 GHz and the impedance bandwidth lower than − 10 dB needs to be expanded. The improved method is to cut off multiple periodic elliptical regular slot edges (RSEs) at top and outer edges of the radiator, thereby transferring the induced current from the outer edges to the inside of antenna, as shown in Fig. 1b. The slotted AVA can increase the effective electrical length, reducing the vertical reflection from the radiator. The RSEs are formed by rotating the center of the ellipses and arranged at equal intervals. After simulation and optimization, it is finally determined to cut off six elliptical RSEs on each side of radiating patch to achieve the goal of antenna miniaturization.

In order to further improve the gain of AVA, a semi-elliptical dielectric lens is loaded at the aperture of the antenna as illustrated in Fig. 1c. Semi-elliptical lens structure can better concentrate the energy in the center of the aperture to achieve the effect of increasing the gain.

Because the RSE structures increase the current length of the radiation patch, the slotted AVA has an excellent performance of 4.5–50 GHz with the ratio bandwidth (*f*_H_/*f*_L_) of 11.1:1 achieving the low frequency reduction from 10 to 4.5 GHz plotted in Fig. 2a. Compared with slotted AVA and lens-loaded AVA shown in Fig. 2b, the maximum gain of lens-loaded AVA reaches 12.7 dBi at 35 GHz, which significantly improves the overall gain of the reference AVA.

**Figure 3:**
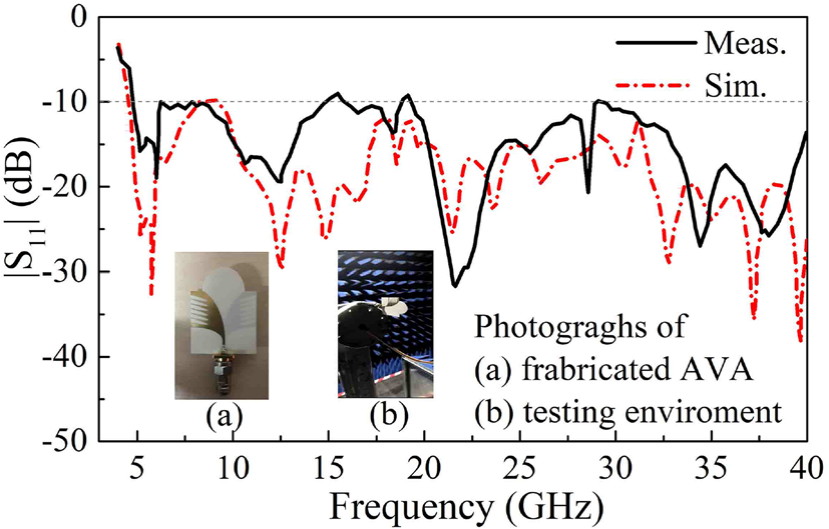
Simulated and measured |S_11_| of AVA.

To verify our simulations, the proposed lens-loaded AVA was fabricated and measured. In Fig. 3, simulated and measured results are in good agreement. The proposed method of combining edges slotting and lens loading which have no conflict with each other and thus we can design a high-gain miniaturized antenna array for UWB communication.

**Figure 4:**
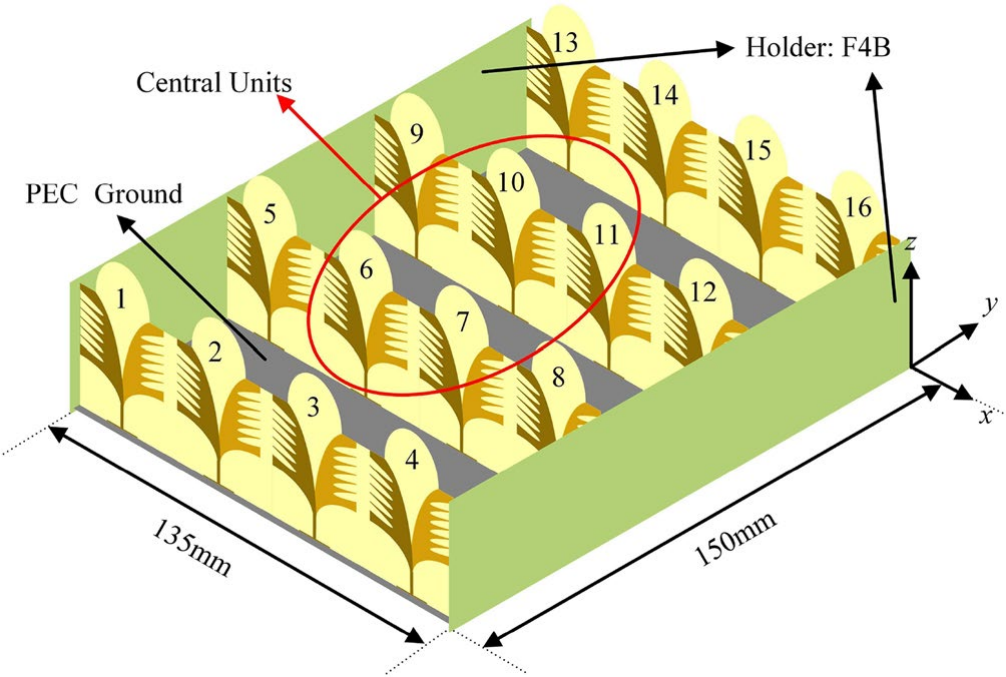
Structure of proposed AVA array model.

Considering the design of AVA array, in order to leave a certain space for loading the metasurface ground, the spacing of *y*-direction should be larger than the *x*-direction. Thereby, the optimized spacing of *x*-direction and *y*-direction is 35, and 50 mm, respectively. Furthermore, in order to fix the array well, F4B (ε_r_ = 2.65) board was used as holders to connect the 1 × 4 array elements. The operating characteristics of the central elements (6, 7, 10, 11) can express the characteristics of the entire 4 × 4 array. The dimension of the entire array is 135 mm × 150 mm, and the structure of the array is illustrated in Fig. 4.

## Design and theory of metasurface

### Design of unit cell

**Figure 5:**
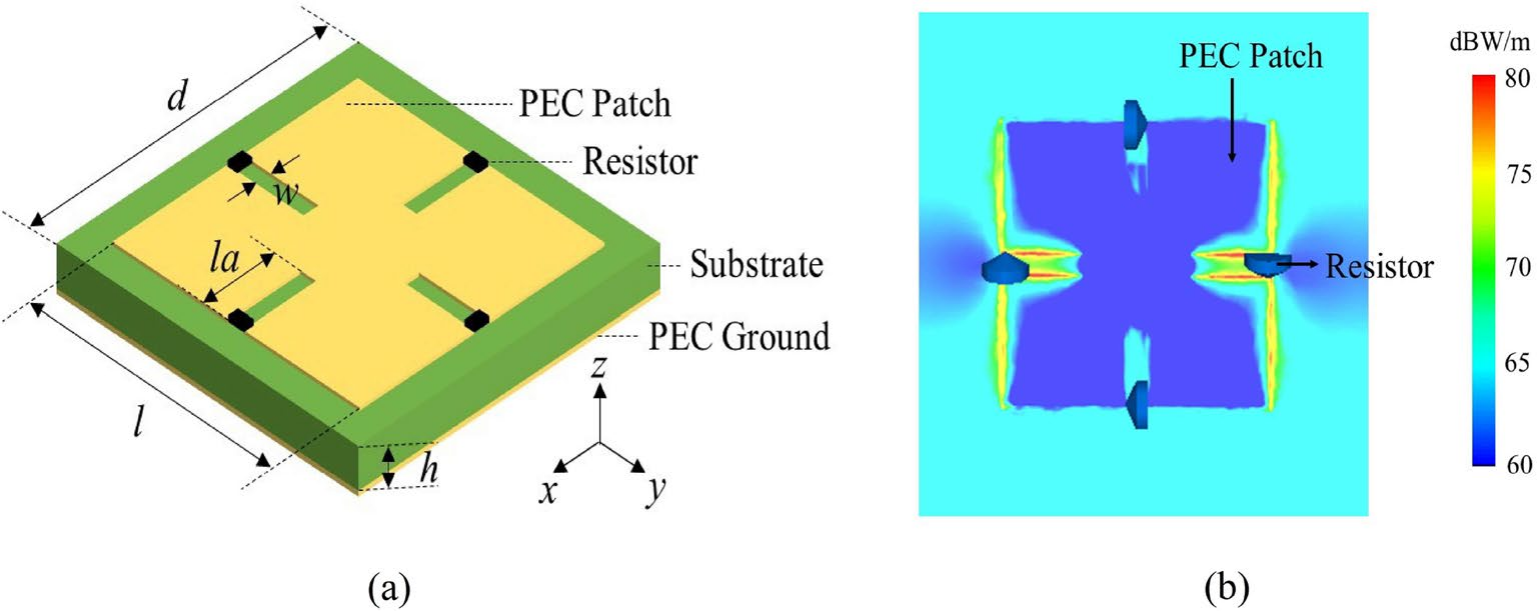
(**a**) Structure model of Minkowski-shaped unit cell. (**b**) Current distribution of the Minkowski-shaped unit cell at 25 GHz (*h* = 0.93 mm, *l* = 5.1 mm).

In this work, we proposed a Minkowski-shaped^20^ metasurface ground reflector for the 4 × 4 AVA arrays. Minkowski geometry was selected because its groove structure of the unit cell is conducive to fabricate and can provide proper space for the welding of resistors. In addition, since the current is the highest in the grooves as shown in Fig. 5b, the resistors can absorb more EM waves. The structure of the unit cell is displayed in Fig. 5a, and the unit cell is composed of two metallic layers separated by substrate F4B (ε_r_ = 2.65) with a thickness of *h*. The top layer consists of a Minkowski-shaped patch, noted as PEC patch with a length of *l*, loaded with four resistors and the bottom one is a PEC ground. The geometrical parameters of the unit cell are optimized as follows: *d* = 9 mm, *la* = *l*/3, *w* = 0.4 mm, and *R* = 150 Ω. Ideally, the reflected EM waves are affected by the loaded resistors and produce changes in amplitude, and the phase of each unit cells will have a large difference by changing the pattern of patch and thickness of substrate.

**Figure 6:**
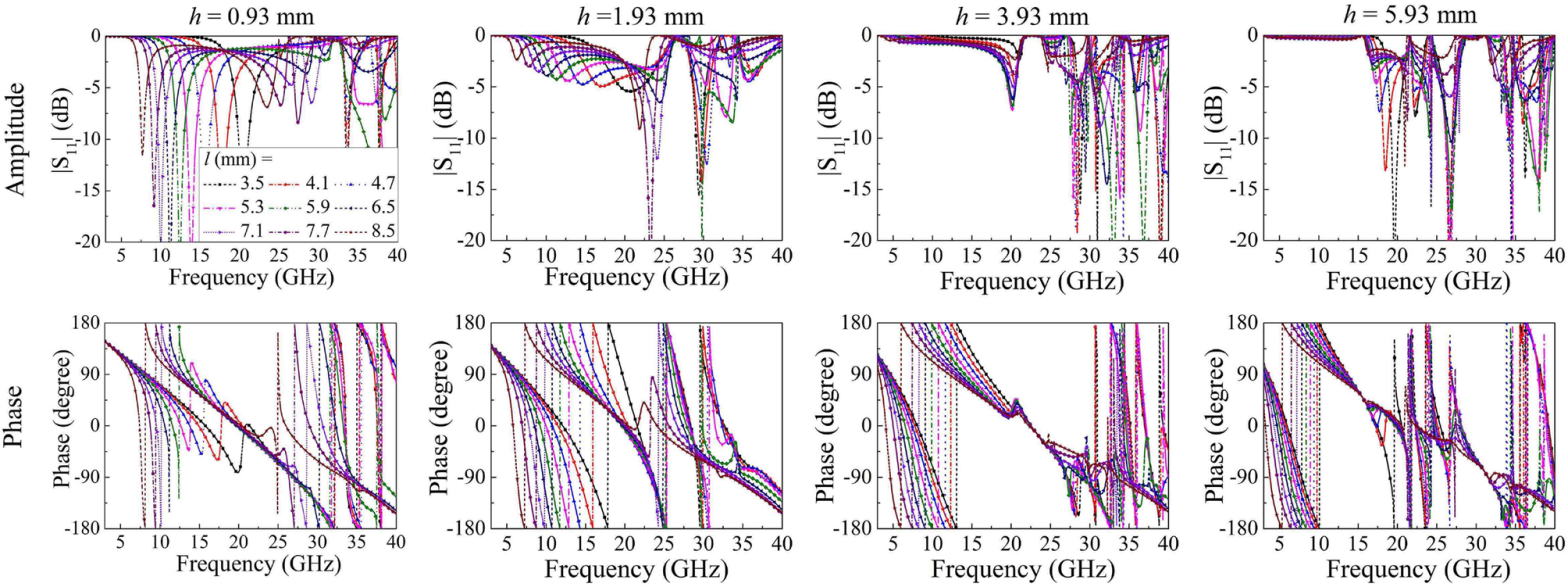
Simulated reflection coefficient of unit cells.

The CST microwave studio is used for simulations in this work, and the reflection coefficient of a part of unit cells are plotted in Fig. 6. The reflection amplitudes below 0 dB can be added to enhance the ability of phase cancellation caused by wave absorption of the resistors. The available phase coverage by tuning the side length of the patch l and dielectric substrate thickness *h* was larger than 180° at frequencies ranging from 5 to 40 GHz. Theoretically, this reflection feature guarantees the possibility of UWB manipulation of EM waves by utilizing MEPC and EM absorption technology.

### Design of metasurface

The interference phenomenon of EM waves is based on the formation of field superposition. When two or more waves from different parts of the metasurface enter the same space, the net amplitude of each point in the space is the sum of the amplitudes of the individual waves. The metasurface is a planar array composed of *M* × *N* lattices. Based on the planar array theory, the scattering field is given^21^,

1 $$E^{S} = EP(\theta ,\;\varphi ) \times AF(\theta ,\;\varphi )$$

*EP* is the pattern function of a tile, which is determined by the structure of the surface, and *AF* is the matrix factor. Under normal incidence, *θ* and *φ* are both 0, and the approximate calculation formula of *AF* can be derived as,

2 $$AF = \sum\limits_{{m = 1}}^{M} {\sum\limits_{{n = 1}}^{N} {\Gamma _{{m,n}} = } } \sum\limits_{{m = 1}}^{M} {\sum\limits_{{n = 1}}^{N} {A_{{m,n}} e^{{j\phi _{{m,n}} }} } }$$

Based on the measurement method of a frequency selective surface, $A_{{m,n}}$ is the amplitude of the lattice reflection coefficient $\Gamma _{{m,n}}$*,* while $\phi _{{m,n}}$ is the phase. The RCS reduction of the metasurface can be approximated^22^,

3 $$\begin{aligned} \sigma _{r} (dB) & = 10\log \left( {\frac{{\sigma _{{meta}} }}{{\sigma _{{PEC}} }}} \right) = 10\log \left( {\frac{{\left| {E_{{_{{meta}} }}^{s} } \right|^{2} }}{{\left| {E_{{_{{PEC}} }}^{s} } \right|^{2} }}} \right) \\ & = 10\log \left( {\frac{{\left| {\sum\nolimits_{{m = 1}}^{M} {\sum\nolimits_{{n = 1}}^{N} {A_{{m,n}} e^{{j\phi _{{m,n}} }} } } } \right|^{2} }}{{(MN)^{2} }}} \right) \\ \end{aligned}$$

Previous RCS researches generally used − 10 dB reduction standard to design the metasurface. However, since maximum radar detection range is negatively correlated with the magnitude of RCS reduction, in order to make our antenna array have better low-scattering characteristics, we use − 15 dB reduction as the standard. Therefore, the RCS reduction of the metasurface requires,

4 $$\sigma _{r} \;({\text{dB}}) \le - 15\;{\text{dB}}$$

On the one hand, the reflection amplitude of the lattice is reduced by loading resistors; on the other hand, phase cancellation is used to mutual compensation and cancellation of multiple EM waves in space. A lattice is composed of 5 × 5 identical small unit cells, and the 3 × 3 different lattices are selected to form the metasurface randomly.

The main purpose is to achieve phase cancellation and EM absorption among the 9 local waves produced by the basic lattices. Particle swarm optimization (PSO)^23^ together with Eq. (3) was used to optimize the side length *l* and layer thickness *h* of unit cells. Before the PSO operation, the reflection amplitude and phase of unit cells need to be precalculated and imported into the algorithm program. According to Eq. (3), the RCS reduction value ($\sigma _{r}$) can be calculated precisely. We finally determine that *l* varies from 3.5 to 8.9 mm with the step width of 0.2 mm and the *h* is 0.93, 1.93, 3.93, and 5.93 mm.

Considering that some frequencies have large $\sigma _{r}$ values and other frequencies have small $\sigma _{r}$ values, the cancellation errors will be generated in the process of operation. The fitness function used to evaluate the performance of predicted RCS reduction is defined as,

5 $${\text{fitness}} = \sum\limits_{{i = 1}}^{K} {s(i)}$$

and

6 $$s(i) = \left\{ {\begin{array}{*{20}c} {1,} & {\quad {\text{if}}\;\;|\sigma _{r}^{i} | < 15\;{\text{dB}}} \\ {0,} & {\quad {\text{if}}\;\;|\sigma _{r}^{i} | \ge 15\;{\text{dB}}} \\ \end{array} } \right.$$

where $\sigma _{r}^{i}$ is the RCS reduction under normal incidence at the *i-*th frequency and the selected frequency we set is a series of discrete numbers varied from 5 to 50 GHz with the step width of 0.1 GHz. *K* is the number of optimized frequency points. The smaller the fitness value it is, the more RCS reduction of this surface will be. The parameters of selected unit cells finally obtained after optimization are shown in Table 1.

**Table 1:** The optimized results of geometrical of selected unit cells.

Unit cell	1	2	3	4	5	6	7	8	9
*h*/mm	0.93	0.93	1.93	1.93	3.93	3.93	3.93	5.93	5.93
*l*/mm	3.5	3.5	8.5	4.5	5.1	6.1	8.1	7.1	7.9

Based on the planar array theory, the backscattered field is only related to the reflection phase, not related to the distribution of elementary lattices under normal incidence. Thereby, lattices are randomly distributed for the diffuse scattering of EM waves, achieving low monostatic RCS without the radiation performance degradation. The dimension of the entire metasurface is 135 mm × 135 mm, so that the array is slightly larger than the metasurface ground reflector at *y*-direction.

**Figure 7:**
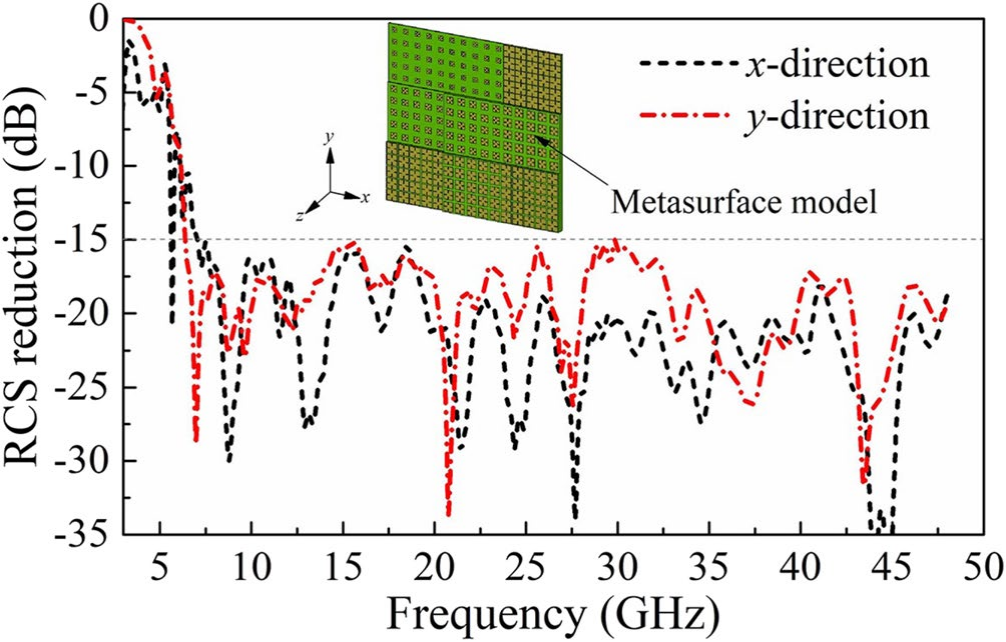
Simulated RCS reduction of metasurface under normal incidence.

A full structure of the metasurface was simulated by the transient solver of CST Microwave Studio. The simulated RCS reduction is illustrated in Fig. 7, which shows that the RCS reduction values from 7.1 to 50 GHz are greater than 15 dB, and values from 5 to 50 GHz are greater than 10 dB with a ratio bandwidth of 10:1. Both polarization results imply that the proposed physical mechanism of MEPC and EM absorption can greatly expand the bandwidth of RCS reduction.

### Simulation of AVA array loaded with metasurface

**Figure 8:**
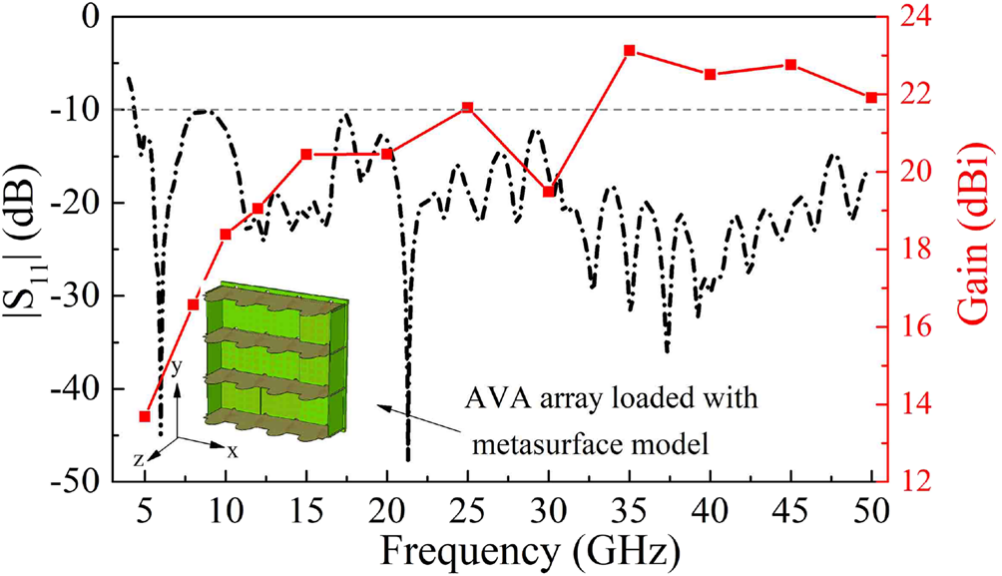
|S_11_| of central element (element 6) and the gain of AVA array loaded with metasurface.

**Figure 9:**
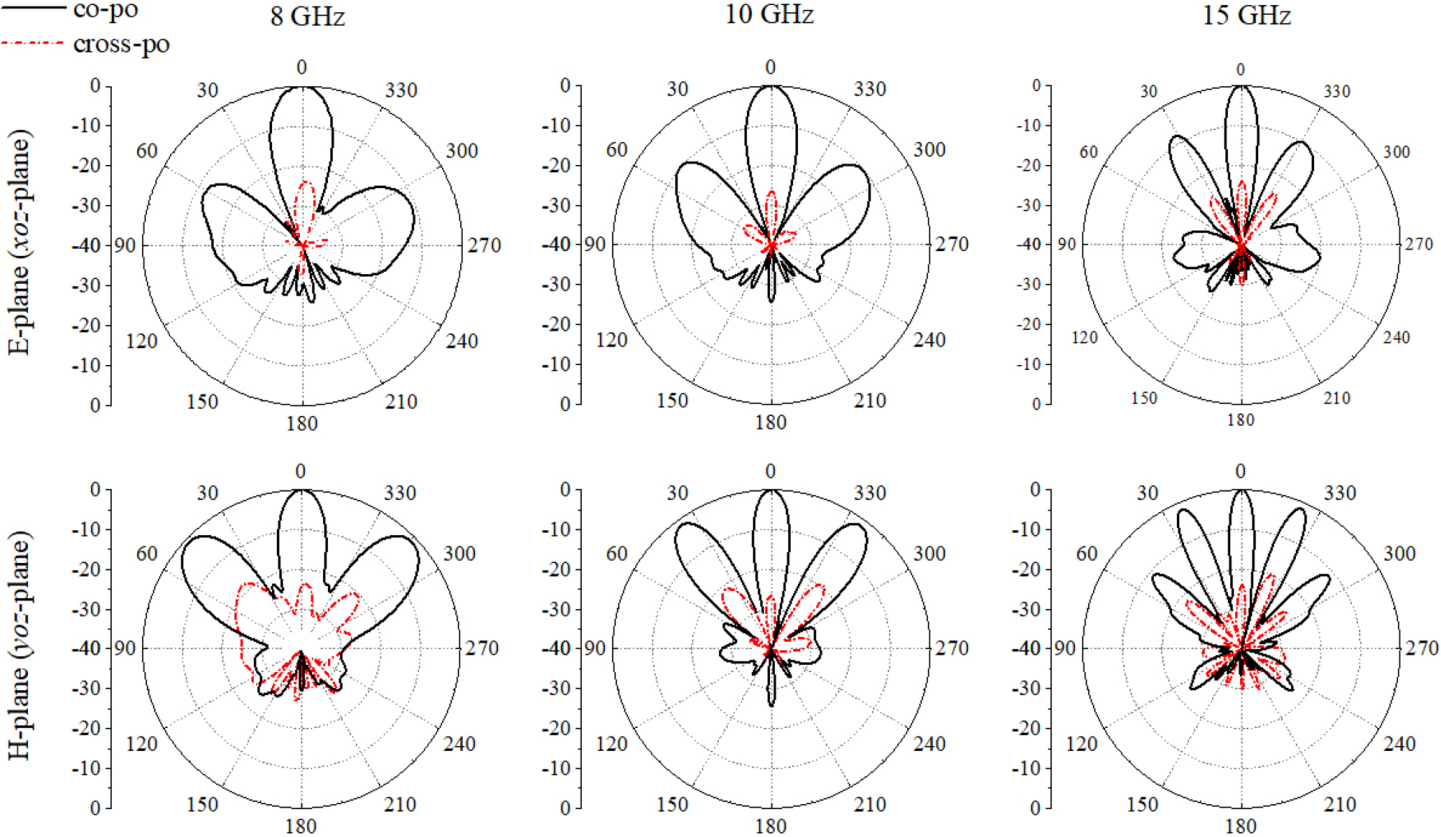
Radiation pattern of AVA array loaded with metasurface.

In Fig. 8, it can be seen that the metasurface has no influence on the reflection characteristics for the operating frequency band of presented antenna array. Figure 9 depicts the radiation pattern of the array at some frequencies, respectively. As observed, the maximum gain of the array reaches 23.5 dBi at 35 GHz, whereas it encounters the grating lobes resulting in a lager decrease at 30 GHz. In this design, the wavelength corresponding to 30 GHz is 10 mm, and the spacing dimension is just integer multiples of it. At this frequency, the number of grating lobes will increase, so the great grating lobes would lead to a serious energy loss in the main lobe. Furthermore, the gain at low frequency band increases more obviously, while it tends to be flat and shows a slow downward trend at high frequency band. Since the distance between the array elements exceeds half wavelength, grating lobes appear in the pattern as the frequency increases, and more grating lobes appear at high frequencies.

Array theory points out that the radiation pattern of an array is the multiplication of the elemental pattern and the array factor determined by the spacing, amplitude, and phase between the elements. However, the large element spacing causes grating lobes occurred at high frequency, thus we should find the methods of eliminating grating lobes. For the uniform periodic arrays, one of the methods is using subarray design diagnostics to suppress undesirable grating lobes^24^, and the other is controlling the amplitude distribution of the peripheral array elements to reduce grating lobes^25^. For the aperiodic arrays, it could be realized by optimizing the arrangement of array elements which can offer bandwidths of many octaves with excellent sidelobe suppression^26,27^.

## Fabrication and measurement

**Figure 10:**
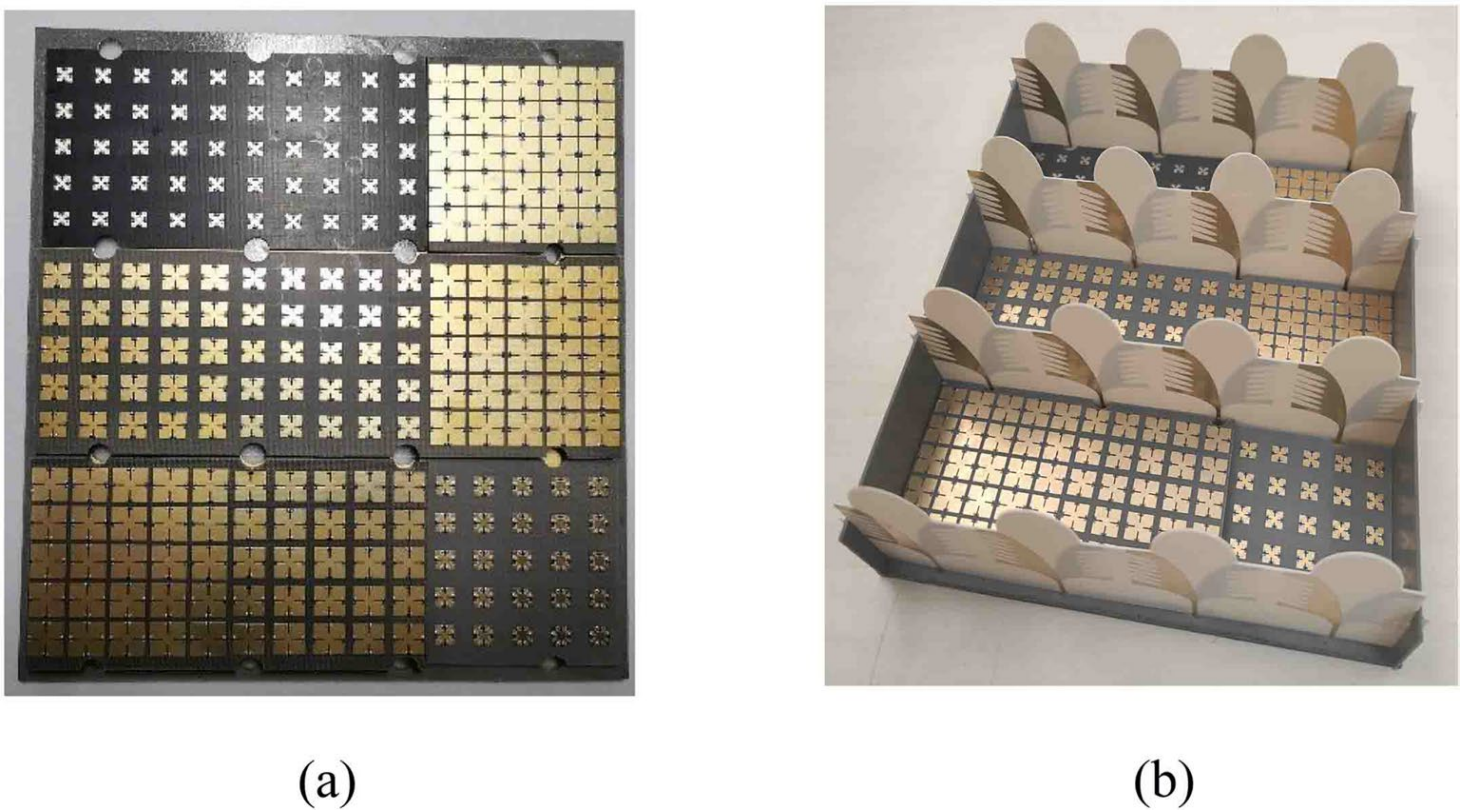
The photographs of the fabricated (**a**) metasurface, (**b**) AVA array loaded with metasurface.

To validate the radiation and scattering characteristics of the presented antenna array, the metasurface and AVA array were fabricated shown in Fig. 10. Due to the limitation of experimental conditions, the test frequency band only can reach up to 40 GHz.

**Figure 11:**
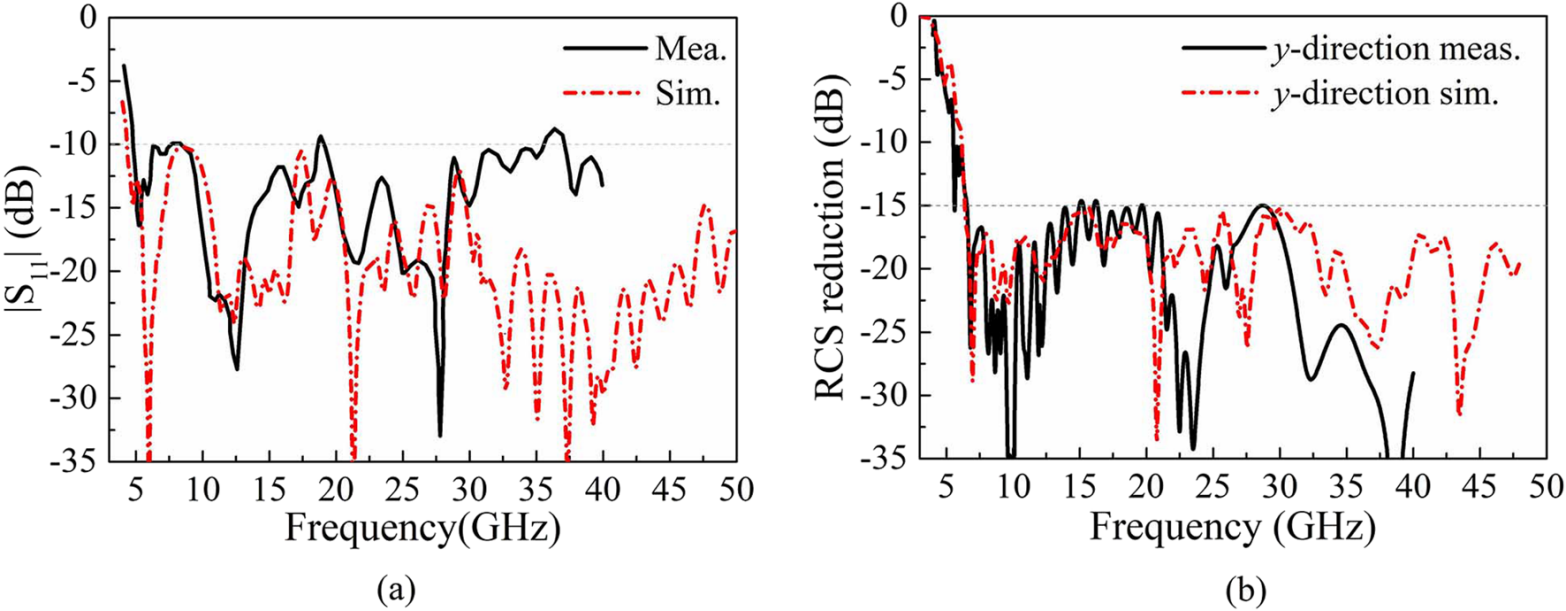
Simulated and measured performance of AVA array loaded with metasurface (**a**) |S11| of central element (element 6). (**b**) RCS reduction.

As shown in Fig. 11a, the simulated |S_11_| of the central element (element 6) are below − 10 dB at the frequency band from 4.5 to 50 GHz, while the measured results are slightly higher than − 10 dB at 36 GHz. Comparatively, there is obvious difference between the simulated and measured |S_11_| at high frequency, which is resulted from the cumulative effects of fabrication, assembly, and testing environment. From the RCS reduction results under normal incidence shown in Fig. 11b, the RCS of the sample is reduced by more than 15 dB within the frequency band of 7.1–40 GHz for *y*-direction polarized incident waves, and the simulated results and measured results are in good agreement in the entire operating frequency band. The measured results have validated that this paper realized a high-performance AVA array with a 10 dB impedance bandwidth from 4.5 to 50 GHz, simultaneously achieved the low observable characteristic with 15 dB RCS reduction nearly covering the operating band.

## Conclusion

A complete design procedure for synthesizing miniaturized UWB low-scattering AVA array has been introduced in this paper. A low-scattering metasurface nearly cover the operating band, which is obtained through phase cancellation and absorption technology by 9 types of AMC structures, is used as the ground reflector to effectively suppress monostatic and bistatic RCS of antenna array. This approach prevents any deterioration in both radiation performance and scattering property. The proposed array realizes a − 10 dB impedance bandwidth is 4.5–50 GHz with a ratio bandwidth (*f*_H_/*f*_L_) of 11.1:1, the maximum gain at 35 GHz is 23.5 dBi, and a 15 dB RCS reduction nearly covering the whole operating band for both polarizations. A prototype has been fabricated and measured to verify the proposed procedure, and measured results are in good agreement with the simulation. The proposed antenna array with high gain, miniaturized size, and UWB low-scattering features can be used in the stealth military.
